# Research progress on albumin-based hydrogels: Properties, preparation methods, types and its application for antitumor-drug delivery and tissue engineering

**DOI:** 10.3389/fbioe.2023.1137145

**Published:** 2023-04-11

**Authors:** Run Meng, Huimin Zhu, Peiying Deng, Minghui Li, Qingzhi Ji, Hao He, Liang Jin, Bochu Wang

**Affiliations:** ^1^ Key Laboratory of Biorheological Science and Technology, Department of Education, College of Bioengineering, Chongqing University, Chongqing, China; ^2^ Sheyang County Comprehensive Inspection and Testing Center, Yancheng, China; ^3^ School of Pharmacy, Yancheng Teachers’ University, Yancheng, China

**Keywords:** albumin, hydrogels, drug delivery, preparation methods, cancer therapy, tissue engineering

## Abstract

Albumin is derived from blood plasma and is the most abundant protein in blood plasma, which has good mechanical properties, biocompatibility and degradability, so albumin is an ideal biomaterial for biomedical applications, and drug-carriers based on albumin can better reduce the cytotoxicity of drug. Currently, there are numerous reviews summarizing the research progress on drug-loaded albumin molecules or nanoparticles. In comparison, the study of albumin-based hydrogels is a relatively small area of research, and few articles have systematically summarized the research progress of albumin-based hydrogels, especially for drug delivery and tissue engineering. Thus, this review summarizes the functional features and preparation methods of albumin-based hydrogels, different types of albumin-based hydrogels and their applications in antitumor drugs, tissue regeneration engineering, etc. Also, potential directions for future research on albumin-based hydrogels are discussed.

## 1 Introduction

**FIGURE 1:**
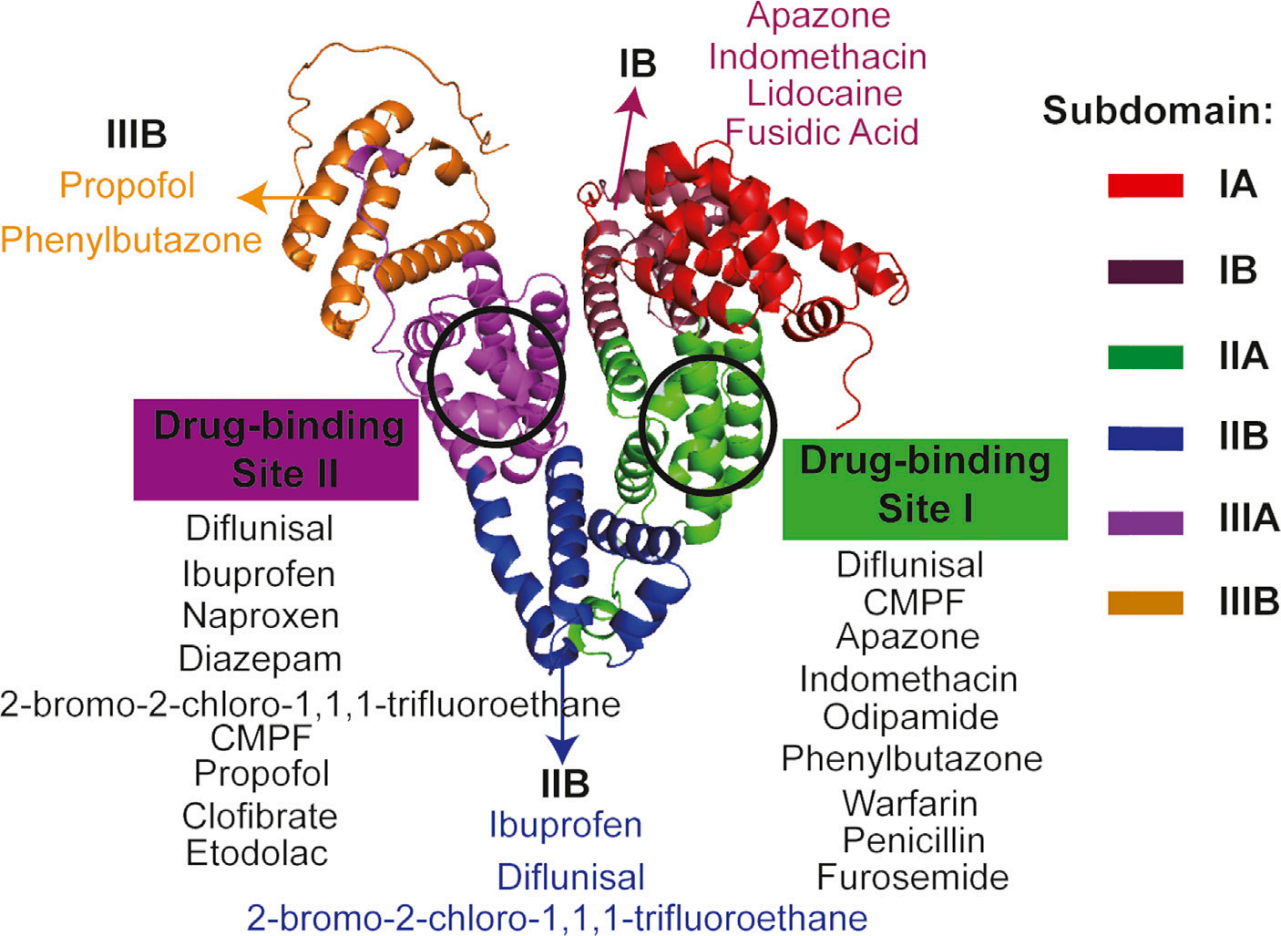
The crystal structure and drug-binding sites of human serum albumin. The crystal structure of human serum albumin is heart-shaped and divided into 3 main regions, each of which can be divided into two sub-regions (Carter et al., 1989; Colmenarejo, 2003). Sudlow et al. first found that drug-binding sites on albumin were concentrated in two regions, site I and site II, respectively (Sudlow et al., 1976). The detailed binding regions for the various drugs are shown in the diagram (Bhattacharya et al., 2000; Petitpas et al., 2001; Colmenarejo, 2003; Kratz, 2008). Full name of CMPF is 3-Carboxy-4-methyl-5-propyl-2-furanpropionic acid.

Albumin is the most abundant plasma protein in human or animal plasma, and human serum albumin (HSA) consists of 585 amino acid residues and has a molecular weight of approximately 67 kDa (Jagdish et al., 2021). Its crystal structure is heart-shaped and has 3 main domains, and each of them can be divided into 2 subdomains (A or B subdomain). Bovine serum albumin (BSA) consists of 583 amino acids and has a molecular weight of approximately 66.43 kDa, whose overall molecular is shaped like a heart and it has the same number of domains or subdomains as HSA (Meng et al., 2022). There are three main functions of serum albumin. Firstly, albumin has a vital role in maintaining osmolarity in the body, and it is more stable and durable than salt-based small molecules in maintaining osmolarity. Secondly, albumin acts as a transport carrier in the body, which can bind a wide range of ions and transport various substances (including many drugs) to various parts of human or animal body. As early as 1976, Sudlow et al. already found that the drug-binding sites on albumin are concentrated in two domains (Figure 1, site I and site II) (Sudlow et al., 1976). And also, albumin can bind toxic substances and transport them to detoxification organs such as the liver. The last, amino acids are the basic building blocks of albumin, which is made up of 20 different amino acids, so albumin is also a major nutrient in the body and it can be used to provide energy in emergency conditions (Bernardi et al., 2020). Thus, albumin is widely used in the biomedical field due to its good degradability, non-immunogenicity, non-cytotoxicity and its multiple drug-binding sites.

Hydrogel is a kind of extremely hydrophilic three-dimensional network-structured gels that swell rapidly in water and can retain a large volume of water in its swollen state without dissolving (Liu J. et al., 2020). Since the invention of hydrogel contact lenses in 1960 (Wichterle and LÍM, 1960), significant research and development of hydrogels for medical applications has been made. For example, in 2013, the research team from Johns Hopkins University reported that they developed a new hydrogel biomaterial that could stimulate the patient’s bone marrow to produce stem cells and grow new cartilage when the new hydrogel biomaterials is injected into small holes in the bone during cartilage repair surgery (Sharma et al., 2013). In clinical trials, 86% coverage of new cartilage was achieved and post-operative pain was greatly reduced. In 2021, scientists from Japan developed a novel hydrogel which can effectively deliver drugs to tumors and respond to changes in temperature and pH in the tumor microenvironment (Komatsu et al., 2021). Recently, researchers from Harvard University reported the use of a tough adhesive hydrogel called Janus Tough Adhesive (JTA) for the treatment and prevention of tendon injuries, showing that JTA exhibited enhanced tissue adhesion and facilitated sliding of surrounding tissue, in addition to serving as a drug delivery system for local agent release (Freedman et al., 2022). There are countless examples like above cases of hydrogel applications in the biomedical field, and this is due to the advantages of hydrogels such as simple preparation process and low cytotoxicity. Though the use of hydrogels in biomedical applications continues to grow and the clinical requirements for hydrogels are also increasing, allergic and inflammatory reactions caused by hydrogels prepared from polymerized polymer compounds have occurred. For example, cases of complications from polyacrylamide hydrogels are frequently reported, and reactions such as inflammation, tissue overgrowth and pain may occur after the injection of polyacrylamide hydrogels (Rauso et al., 2019; Ding et al., 2022; Kose et al., 2022). Adverse reactions caused by other polymer hydrogels also occur from time to time, such as Burnshield^®^, silicone hydrogel, Miragel^®^ and so on (Robertson and Cavanagh, 2005; Storan et al., 2016; Albalawi et al., 2022). Due to the inherent chemical and physical cross-linking network, the traditional hydrogel is not biodegradable, which can also result in serious environmental pollution (Guo et al., 2023). In addition, traditional hydrogels represented by polymer hydrogels have the disadvantage of uncontrollable mechanical properties (Li et al., 2021; Elangwe et al., 2022). It is due to these defects in polymer hydrogels that researchers are still seeking hydrogels with better biodegradability, non-immunogenicity and excellent mechanical properties.

Proteins, a natural material with good biocompatibility, have naturally attracted the attention of researchers (Hughes et al., 2022). Proteins such as albumin, ferritin, silk proteins, keratin, mucin and many more have been used in the preparation of hydrogels, and these protein-based hydrogels exhibit various properties and can be used in a variety of applications (Silva et al., 2014; Hong et al., 2020; Kim et al., 2021; Wang R. et al., 2022; Ye et al., 2022). Among these proteins, albumin, as a plasma-derived protein, has many advantages like the ones we have introduced before. Obviously, it is also increasingly used for the preparation of various novel hydrogels. The methods of preparation, detailed functions and applications of these albumin-based hydrogels are also different. To date, there are only a few articles that systematically describe the research progress of albumin hydrogels. However, these reviews have different emphases. For example, the review written by Kong et al. provides a detailed and insightful summary on albumin-based hydrogels, however, their focus is on summarizing the use of albumin hydrogels for personalized biomaterials and 3D printing (Kong et al., 2023). The review of Ong et al. is a mini review, which reports current literature and critically discusses the synthesis, mechanical properties, biological effects and uses, biodegradability and cost of albumin-based hydrogels as of 2019, especially the application of tissue engineering and regenerative medicine (Ong et al., 2019). In this review, we highlight the application of albumin-based hydrogels in antitumor drug delivery and tissue engineering. Besides, in order to provide a relatively comprehensive understanding of hydrogel research, this paper summarizes the properties, types, and recent research progress of albumin-based hydrogels in detail. Potential research directions are also summarized on the basis of problems encountered in the experiments and extensive literature reading.

## 2 Properties and preparation methods of albumin-based hydrogels

### 2.1 Properties

Hydrogels prepared using albumin as the basic unit have excellent properties, such as good mechanical properties, biocompatibility, biodegradability, and so on (Amdursky et al., 2018; Xia et al., 2021). These properties make albumin-based hydrogels promising for applications in the biomedical field. To date, due to their excellent properties, albumin-based hydrogels have been used in a wide range of applications including drug delivery, tissue regeneration engineering, diagnostics, cell transplantation and more (Chander et al., 2021; Mahdipour and Mequanint, 2022; Zhang et al., 2023). In this section, we summarize in detail the three main properties of albumin-based hydrogels to increase the knowledge of their advantageous properties.

#### 2.1.1 Mechanical strength

The excellent mechanical properties of albumin-based hydrogels are mainly due to the controllable-mechanical properties (Tensile strength is in the range from serval to hundreds (kPa) and compressive strength is in the range from hundreds kPa to tens MPa, which can meet the needs of different biomedical applications, for example, soft albumin-based hydrogels can be used as injectable hydrogels, a medium-hard albumin-based hydrogel can be used as a cosmetic filling material and hard albumin-based hydrogels can be used as bone regeneration scaffolds.), which vary from different methods of preparing albumin-based hydrogels (Arjama et al., 2021; Kong et al., 2023). Researchers often prepare albumin-based hydrogels with specific mechanical-properties depending on the application requirements (Ong et al., 2019). The hydrogel prepared by Xia et al. using bovine serum albumin has better elasticity, greater mechanical strength and also better self-healing properties, whose storage modulus reaches ∼10 kPa and loss modulus reaches ∼1 kPa (Xia et al., 2021). Even at a concentration of 10 mg/mL of this hydrogel, the survival rate of L929 cells remains above 85%, so the BSA-based hydrogel shows better biocompatibility compared to conventional polymer hydrogels. Besides, in antibacterial tests, 6 mg/mL BSA-based hydrogels showed a strong bactericidal effect, there is almost no bacterial growth after the addition of the hydrogel, the inhibition rate reached ∼99.9% for *E. coli* and *S. aureus*, this finding suggests that the BSA-based hydrogel constructed by Xia et al. has promising applications in the field of antimicrobial activity. The albumin-based hydrogel exhibited good anti-microbial activity. In 2016, a research team from Tsinghua University prepared a new hydrogel using bovine serum albumin, which has good deformability and can be injected through a narrow needle (Sun and Huang, 2016). *In vitro* drug release studies have shown that this albumin-based hydrogel has a good sustained drug-release. Natural protein-based hydrogels have excellent biocompatibility, however, most of them are weak or brittle. In 2020, Lu et al. reported an albumin-based hydrogel with high compressive and tensile strength, possessing rapid self-recovery and excellent fatigue-resistance (Lu et al., 2020). During the preparation of albumin-based hydrogels, ethanol is also often used as a trigger for the denaturation and subsequent gelation of serum albumin. For example, Seyed et al. used ethanol to reduce the proportion of strongly bound FAs in BSA-based hydrogels by 52% and induced a maximum storage modulus of 5,000 Pa in BSA-based hydrogels (Arabi et al., 2020). The excellent mechanical properties of albumin-based hydrogels are not only reflected in the high or low mechanical strength, but also in the rapid self-healing (Wang J.-T. et al., 2022). Wang et al. demonstrated through tensile and compression tests that the rapid self-healing properties of the BSA complex hydrogel constructed by themselves, which is able to meet the needs of some practical applications, such as wound healing dressings, tissue scaffold materials, beauty products and so on (Ganesan and Choi, 2016; Kharaziha et al., 2021; Sun et al., 2021).

#### 2.1.2 Biocompatibility

Albumin is mainly derived from human or animal serum, there are almost no heterologous groups on its surface and the protein surface is generally hydrophilic, so albumin does not cause any immune reaction and it has better biocompatibility. Therefore, hydrogels constructed with albumin as the basic structural unit also have good biocompatibility. The better biocompatibility of albumin-based hydrogels is mainly reflected in their low cytotoxicity, non-immunogenicity and better ability to promote cell or tissue growth (Chen et al., 2020). The biocompatibility of albumin-based hydrogels is often demonstrated by *in vitro* and *in vivo* experiments. For *in vitro* experiments, the evaluation is generally done mainly by cytotoxicity, and for *in vivo* experiments, it is mainly evaluated by whether it causes an inflammatory response, whether there is immune rejection, etc. The biocompatibility study of BSA-based hydrogel by Liu et al. showed that L929 cells maintain a survival rate close to ∼100% at 5 mg/mL BSA-based hydrogel, and there are almost no dead cells (Liu et al., 2021). Raja et al. constructed pH- and redox-sensitive albumin-based hydrogels, and it has good stability, rheological properties and compressive strength. Cellular assays and analysis of blood samples revealed the low cytotoxicity (The survival rate of NIH 3T3 cells exceeded 95% at albumin-based hydrogel of 450 μM) and non-immunogenicity of this albumin-based hydrogel (Raja et al., 2015). Chen et al. obtained highly biocompatible albumin hydrogels by a heat treatment method. *In vivo* experiments with immunoreactive mice showed that the subcutaneously injected albumin-based hydrogel was completely degraded with negligible acute inflammatory response, indicating its excellent *in vivo* biocompatibility (Chen et al., 2016). *In vitro* cellular assays have also confirmed the low cytotoxicity of this albumin-based hydrogel. The excellent biocompatibility of albumin-based hydrogels provides the basis for their use in biomedical field, particularly in the fields of tissue regeneration engineering and drug delivery.

#### 2.1.3 Degradability

In many applications where hydrogels are used as drug delivery systems, the drug is slowly released during the gradual degradation of the hydrogel. For example, a citrate-based hydrogel can improve cardiac repair after myocardial infarction by continuously releasing the encapsulated growth factor (Mydgf) during gradual degradation (Yuan et al., 2019). An aminoglycoside hydrogel with adjustable hydrogel-degradation enables on-demand release of antibiotics and demonstrates good antibacterial activity (The survival rate for *E. coli*, *Pseudomonas aeruginosa*, *S. epidermidis* and *S. aureus* are all below 5%.) (Hu et al., 2017). In view of this, the biodegradability of hydrogels is very important for their clinical application, which avoids the pain caused to patients by secondary surgery. While, hydrogels made from albumin have excellent biodegradability properties. Albumin is derived from human or animal plasma, which is naturally degradable and has a half-life of approximately 14 days (Meng et al., 2022). So, hydrogels prepared using albumin can also degrade under physiological conditions. For example, the albumin-based hydrogel developed by Zhao et al. shows accelerated degradation under light conditions at 37°C, and the albumin-based hydrogel degrades to less than half its volume within 24 h (Zhao et al., 2019). Researchers from France constructed an albumin-based hydrogel that shows significant degradation after 24 h of continuous stirring in a solution containing thermolysin, and the degradability of this albumin-based hydrogel is further confirmed by the observation of scanning electron microscopy (Deneufchâtel et al., 2018). In 2021, Mao et al. reported a biodegradable albumin-based hydrogel with adjustable biodegradability, which allows precise drug release and has good prospects for applications (Mao et al., 2021). Researchers from University of Washington used 3D printing to produce a BSA-based hydrogel that also has good biodegradable properties, and this albumin-based hydrogel is expected to be mass-produced through 3D printing technology (Smith et al., 2020). Obviously, composite albumin-based hydrogels have also been prepared using albumin and other compounds. The composite hydrogel obtained by Zhang et al. using albumin and hyaluronic acid not only exhibits excellent mechanical properties but also possesses good degradability. In addition, the albumin-composite hydrogel also demonstrated the ability to bind to hydrophobic substances (Hájovská et al., 2020). The degradability of albumin-based hydrogels provides a solid basis for their use in tissue engineering, particularly as functional scaffold materials (Yuan et al., 2020).

### 2.2 Preparation methods

Many factors can cause changes in the structure and properties of protein hydrogels under certain conditions, such as temperature, mechanical forces, light, pH value and so on (Navarra et al., 2016; Sharma and Pandey, 2021). Thus, there are a number of specific methods for preparing albumin-based hydrogels, however, in terms of broad categories, albumin hydrogels are divided into two main methods: chemical crosslinking and physical crosslinking (Huang et al., 2017). Depending on the application requirements, the hydrogel preparation method which is chosen would vary.

#### 2.2.1 Chemical crosslinking

The preparation of albumin-based hydrogels by chemical crosslinking is essentially the formation of covalent bonds between molecules. Unlike physical hydrogels, chemical hydrogels are not reversible due to the three-dimensional lattice structure connected by covalent bonds. The structure and properties of albumin-based hydrogels can be achieved by changing the initiator, crosslinker and chain transfer agent to control the kinetic action of the polymerisation reaction. For example, Sun et al. obtained albumin-based hydrogels cross-linked by disulfide bonds under acidic conditions by redoxing BSA (Sun and Huang, 2016). Researchers from Germany used ethanol as a trigger for the denaturation and final gelation of serum albumin, and they explored the space of conformational and physical properties of albumin-based hydrogels when they are induced by ethanol, the results showed that the use of ethanol is desirable for certain hydrogel properties at the nano- and macro-scale (Arabi et al., 2020). Zhou et al. constructed a bioglass-activated albumin hydrogel for wound healing, and the hydrogel was formed mainly by cross-linking between HSA and succinimidyl succinate modified polyethylene glycol (PEG), and the researchers regulated the gelling time of the composite albumin hydrogel by varying the amount of bioglass, which gave the hydrogel good injectability (Zhou et al., 2018). Despite the simplicity of the chemical cross-linking process and the fact that it is one of the most commonly used methods for preparing hydrogel, it still has its drawbacks (Rodin et al., 2021; Hu et al., 2022). The main disadvantage of the chemical cross-linking method for the preparation of albumin-based hydrogels is the possibility of residual toxic chemicals or reaction by-products, which to some extent limits the use of hydrogels obtained by the chemical cross-linking method (McClements, 2017; Van Hoorick et al., 2019).

#### 2.2.2 Physical crosslinking

The physical cross-linking method refers to the formation of differential zones, such as microcrystals, micelles, helices and complex cross-linking zones, through non-covalent bonds such as Van Der Waals forces, hydrogen bonding, electrostatic attraction and hydrophobic interactions, which ultimately cross-link the molecular chains of polymer to obtain a hydrogel (Basu et al., 2016; Mantooth et al., 2019). The physical cross-linking method does not use organic cross-linking agents, which can maintain good biocompatibility and allow the physical cross-linking point to change with changes of environmental parameters. The albumin-based hydrogel prepared by physical cross-linking method is reversible and soluble, and the physical cross-linking method is widely used in the preparation of hydrogels because it is a much simpler process (Nie et al., 2019; Xue et al., 2022). A kind of BSA composite hydrogel was prepared by Guo et al. *via* physical cross-linking method (non-covalent interactions), this composite hydrogel is a kind of adhesive hydrogels (Adhesive strengths of ∼350–390 kPa have been achieved.) and was shown to have good mechanical properties by shear testing, so this albumin composite hydrogel has shown some promise in biomedical applications such as tissue adhesives, wound dressings and drug delivery (Guo et al., 2022). Physical crosslinking is also often used in conjunction with chemical crosslinking to prepare albumin-based hydrogels. As in the work of Lu et al. that we described earlier, a composite albumin hydrogel was prepared by combining physical cross-linking with chemical cross-linking (The physical crosslinking method used in this research is heat-denaturing crosslinking, while the chemical crosslinking method is mainly *via* APS/Ru(II) under white light.). The obtained hydrogel exhibited excellent mechanical properties (The hydrogel shows a high compressive strength of ∼37.81 MPa and a tensile strength of ∼0.62 MPa under optimal conditions.) (Lu et al., 2020).

It is worth noting that the ordered amyloid structures formed *in vitro via* chemical crosslinking or physical crosslinking are ideal scaffolds for many biomedical applications. For example, a network of amyloid fibrils under specific stimuli can form stable hydrogels, and Khanna et al. used BSA as a model amyloidogenic protein to obtain thermally-induced hydrogels which display tunable sol-gel-sol transitions spanning over minutes to days, the hydrogel could be used for topical drug delivery (Khanna et al., 2020). Protein-amyloid fibrils form hydrogels more readily than their monomers as well as form stable interfaces and exhibit higher antioxidant activity. Most proteins can self-assemble to form amyloid fibrils under appropriate processing conditions, and albumin is no exception (Knowles and Mezzenga, 2016; Eisenberg and Sawaya, 2017; Cao and Mezzenga, 2019). The unique structure of amyloid fibrils gives them extraordinary physical, chemical and biological properties, further defining their potential for applications in drug delivery and tissue engineering (Xuan et al., 2021). The detailed properties of amyloid fibrils and its use in albumin-based hydrogels are shown in Table 1

**TABLE 1:** The detailed properties of amyloid fibrils and its use in albumin-based hydrogels.

Type	Specific properties	Description
Physical properties	Large aspect ratio	Amyloid fibrils are typically a few nanometres wide (d), while having a length (L) of several tens of micrometres, so it has a large aspect ratio (L/d) (Chiti and Dobson, 2017; Cao and Mezzenga, 2019). Scanning electron microscopic observations of the BSA-based hydrogel constructed by Chiang et al. showed that it was also composed of an amyloid fibrous structure, which also showed a large aspect ratio (Chiang et al., 2020). Meanwhile, this BSA-based hydrogel exhibits a slow-release effect due to its porous structure and hybrid siloxane bridges
	High Young’s modulus	The presence of a densely ordered network of hydrogen bonds in the crossed beta structure, similar to the crystalline region of a polymer, gives the fibrils a Young’s modulus of 2–4 GPa and makes them one of the hardest protein materials (Adamcik et al., 2012). A BSA-based conductive hydrogel prepared by physical cross-linking has excellent mechanical properties (1.61 MPa elastic modulus, 17.66 MJ/m^3^ toughness, and 5.36 MPa tensile stress) and shows good promise for biomedical applications (Xu et al., 2023)
	High surface hydrophobicity	The fibrillation process often involves the unfolding of the internal structure of the protein, leading to the exposure of more hydrophobic amino acids (Mohammadian et al., 2019; Zhou et al., 2020). The HSA hydrogel was constructed by Ana et al., which has a good hydrophobic drug-binding pocket and can be loaded with various substances (especially for hydrophobic drugs) (Vesković et al., 2022)
	Controlled flexibility	Nanofibers with different flexibility, including flexible, semi-flexible and rigid dimensions, can be prepared by simply adjusting the fibrillation conditions, such as pH, ionic strength, protein concentration, etc (Cao and Mezzenga, 2019; Jirkovec et al., 2021). Injectable redox albumin-based hydrogel with *in-situ* loaded dihydromyricetin constructed by Deng et al., which shows excellent self-healing property, elasticity and biocompatibility and can be used for drug delivery (such as dihydromyricetin) (Deng et al., 2022)
Chemical properties	Highly tolerant of the environment	Compared to protein monomers, amyloid fibrils are more tolerant of extreme environments such as acid, heat and enzymes. Which is due to the low free energy of the crossed *ß*-structure and the regular and dense structure (Adamcik and Mezzenga, 2018; Babinchak and Surewicz, 2020). A kind of hydrogel capsules based on HSA developed by Chen et al., which is well tolerated in acidic environments and acts better as a protective layer for MRI probes, providing the prerequisite for more accurate results in complex physiological environments (Xu et al., 2021)
	More active sites	Fibrillation exposes more functional groups in the protein, enhancing their ability to interact with drug molecules. In addition, the ordered *ß*-sheet arrangement enhances the synergistic effect between amino acids (Bhattacharya et al., 2014; Mohammadian and Madadlou, 2016; Maciążek-Jurczyk et al., 2020). There are numerous drug-binding sites on albumin, two main drug-binding sites for albumin are Sudlow site I and Sudlow site II, and albumin can bind more drugs when these two sites are sufficiently exposed (Rong et al., 2022; Bercea et al., 2023)
Biological properties	Low allergenicity	Protein amyloid fibrils are derived from natural proteins, which are more biocompatible and have a lower probability of causing allergic reactions (Willbold et al., 2021). Amyloid fibrils can be used as building blocks for novel albumin-based hydrogel materials that are biocompatible, low cost and hypoallergenic (Diaz and Missirlis, 2022)
	High biological activity	Compared to protein monomers, some amyloid fibrils have stronger antioxidant and antibacterial activity. Some amino acids have high antioxidant activity, such as cysteine, methionine, tryptophan, tyrosine and phenylalanine, and these amino acids are also often involved in the formation of amyloid fibrils. Tryptophan and phenylalanine are involved in protein fibrillation through *p*-π stacking (Makin et al., 2005). Albumin amyloid fibrils still retain more bioactive sites of natural albumin and can be used as drug carriers to bind a wide range of biomolecules and drugs (Ong et al., 2019; Kong et al., 2023)
	High cell permeability	The ability of nanoparticles to penetrate cell membranes is closely related to their size, shape and charge, and the rod-like structure of amyloid fibrils facilitates cellular phagocytosis (Albanese et al., 2012; Sokolova et al., 2020)

## 3 Hydrogels based on different types of albumin

Compared to HSA, BSA is more frequently used in the preparation of albumin-based hydrogels because it is more readily available and less expensive than HSA (Ong et al., 2019). Other types of albumin, such as ovalbumin, murine albumin, equine serum albumin, leporine serum albumin and caprine serum albumin, have been studied very infrequently, let alone used in the preparation of hydrogels (Bujacz et al., 2014; Gałęcki and Kowalska-Baron, 2016; Jia et al., 2021). Meanwhile, the properties and applications of hydrogels prepared from different types of albumin vary slightly, and here we present a summary of the hydrogels prepared from different types of albumin.

### 3.1 HSA-based hydrogels

HSA-based hydrogels are often found in the applications of drug delivery because of the multiple drug-binding sites they possess. Vesković et al. constructed a hydrogel drug-depot using HSA, which can be used for sustained release of a highly cytotoxic modified paullone ligand bearing a TEMPO free radical (HL), so it demonstrated excellent drug-delivery potential (Vesković et al., 2022). In 2014, a hydrogel constructed from HSA by Gao et al., which can effectively carry hydrophobic drugs such as ibuprofen, paclitaxel and dexamethasone. *In vitro* drug release study, all three model drugs had an effective sustained release time of 140 h. Thus, the HSA-based hydrogel demonstrated the excellent potential as a macroscale delivery system, and it is expected to be used in a wide range of biomedical applications including antitumor, tissue engineering, plastic surgery and so on (Gao et al., 2014a). HSA-based hydrogels are also not limited to drug delivery, and they are also used in sensors, reducing inflammatory responses, *in vivo* imaging, wound healing, bone repair, etc. As albumin can also bind to numerous ions, such as Ca^2+^, Zn^2+^, etc (Konopka and Neilands, 1984; Lu et al., 2008). Thus, researchers from University of Pittsburgh constructed a hydrogel sensor using HSA, and it can be used to monitor protein-ionic species binding (Cai et al., 2014). Xu et al. constructed a HSA-based hydrogel which reduces random flap necrosis by attenuating the apoptotic and inflammatory effects of vascular endothelial cells, and its effectiveness has been well proven at the cellular and animal level (Siemiradzka et al., 2021). In 2021, Xu et al. constructed a composite hydrogel based on HSA, which can be used for gastric pH monitoring *in situ* magnetic resonance imaging (MRI) (Xu et al., 2021). This HSA-composite hydrogel has been validated in a rabbit model, fully confirming the capability of this HSA-composite hydrogel to identify abnormal gastric pH. Thus, the construction strategy of HSA-composite hydrogel with well biocompatibility is expected to be an effective tool for *in situ* anti-interference MRI of gastric pH in future clinical applications. In the application of promoting wound healing, Xu et al. constructed a bio-glass hydrogel using HSA that was effective in promoting wound healing (Zhou et al., 2018). Scientists from Israel constructed a composite hydrogel using HSA that can significantly repair tibial defects, and it is effective in accelerating the bridging of tibial bone defects (Kossover et al., 2020).

### 3.2 BSA-based hydrogels

BSA is more widely used in the preparation of albumin-based hydrogels because it is widely available and relatively inexpensive. Like HSA-based hydrogels, BSA-based hydrogels also have many applications. Researchers from Australia used BSA to construct a reversible pH-responsive BSA-based hydrogel, and the aim of this study was to design an efficient pH-responsive bio-diagnostic and drug delivery system (Raghuwanshi et al., 2020). Yang et al. constructed an albumin-based hydrogel sensor using BSA and the prepared biosensor can quantify the concentration of IgG in complex human serum with high sensitivity (Yang et al., 2022). Some researchers also used the BSA-based hydrogel as a substrate for enzyme immobilisation and demonstrated their potential application through *in vitro* biochemical experiments (Belgoudi and Fortier, 1999). BSA-based hydrogels have also shown good application in cancer treatment and luminescent imaging. Zhao et al. reduced the harmful side-effects of metals by using BSA-based hydrogels as a delivery strategy, and this strategy had been proven to be effective in some experimental studies (Zhao et al., 2019). BSA is also frequently used in the preparation of injectable hydrogels, and BSA-based hydrogels show greater potential for the application of injectable hydrogels (Lee et al., 2014; Bai et al., 2019; Liu W. et al., 2020). Also, BSA-based hydrogels have shown good results in promoting wound healing. The BSA-based injectable hydrogel constructed by Zhang et al. is better bonded, biocompatible and biodegradable. These properties have been proven through a series of experiments *in vivo* or *in vitro*, and the BSA-based hydrogel shows excellent potential in heat-sensitive bioconjugate hydrogels in wound healing and tissue regeneration without the need for any other biological factors or inorganic nanoparticles (Phan et al., 2021). Khanna et al. also prepared a heat-responsive BSA-based hydrogel, which was shown to be well suitable for topical drug delivery through a series of *in vitro* and *in vivo* experiments (Khanna et al., 2020). In addition, BSA-based hydrogels have also been investigated for applications in tissue regeneration, the preparation of thermosensitive or temperature-sensitive hydrogels, the prevention of post-operative adhesions and so on (Arabi et al., 2018; Katarivas Levy et al., 2019; Mao et al., 2021; Yoon et al., 2021).

### 3.3 Hydrogels based on other types of albumin

Other types of albumin, such as ovalbumin, murine albumin, equine serum albumin, leporine serum albumin and caprine serum albumin, are used less frequently in the preparation of albumin-based hydrogels. However, the crystal structure of these types of albumin has been elucidated (Bujacz, 2012; Bujacz et al., 2017; Czub et al., 2020; Zielinski et al., 2020). As early as 1992, modeling of gelation with egg white proteins had been attempted by Hsien et al., although ovalbumin is not the only protein component of egg whites, it still makes up a significant proportion of the total (Hsien and Regenstein, 1992). In 2020, researchers from China had constructed a strongly adsorbent hydrogel using ovalbumin, and the ovalbumin-based hydrogel may be a promising adsorbent for the treatment of DFS-contaminated wastewater (Godiya et al., 2020). Researchers from Singapore produced a composite hydrogel using ovalbumin, which has shown to deliver vaccines consistently *via* a series of experiments in a mouse model of lymphoma metastasis (Lee et al., 2019). The reason why ovalbumin is rarely used for hydrogel preparation may be the relatively high cost and low feasibility of isolating and purifying ovalbumin. Secondly, the biocompatibility of ovalbumin is not very good compared to serum-derived albumin, and there are several studies that have reported adverse reactions caused by ovalbumin (Galvão et al., 2017; Shin et al., 2017; Yan et al., 2020). The last is that the molecular conformation of ovalbumin is very different from that of HSA and BSA, and no studies have reported a clear drug-binding site for it, therefore, which is one of the reasons why it is rarely used to prepare albumin-based hydrogels for drug delivery (Owuor et al., 2018; Zheng et al., 2022). While, for other types of albumin, such as murine albumin, equine serum albumin, leporine serum albumin, caprine serum albumin and so on, the source of raw material is relatively less, so it is easy to understand that it is hardly used for the preparation of albumin-based hydrogel.

## 4 Two key applications of albumin-based hydrogels

**FIGURE 2:**
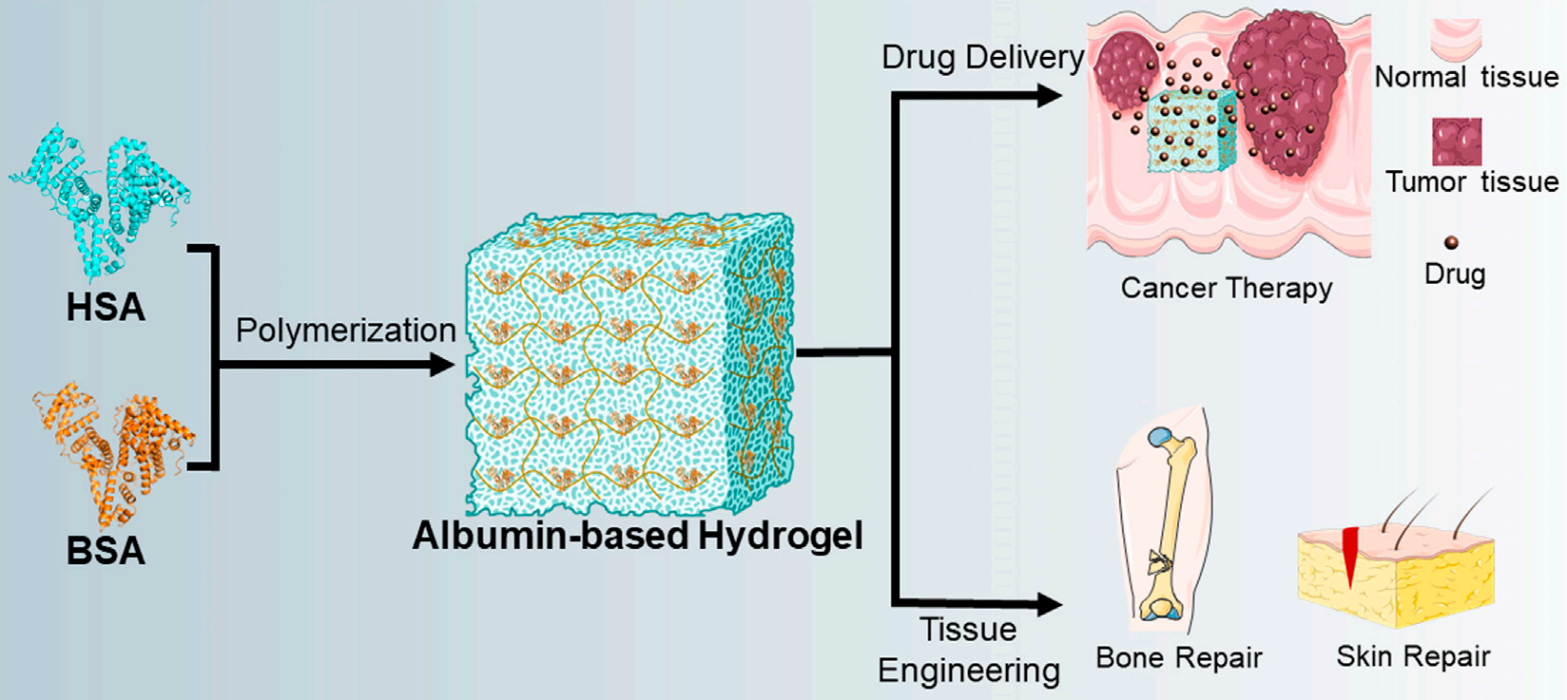
Diagram of the preparation of albumin-based hydrogels and their applications. In general, BSA is used more frequently in the preparation of albumin-based hydrogels than HSA. Two main applications for albumin-based hydrogels are drug delivery and tissue engineering, and detailed applications include the delivery of anti-tumor drugs, bone repair, skin repair, etc.

There are two most important applications of albumin-based hydrogels, the first is drug delivery and the second is tissue regeneration engineering (Ong et al., 2019; Sabaa et al., 2019; Mahdipour and Mequanint, 2022). Meanwhile, albumin-based hydrogels also have many different applications in the field of drug delivery, for example, hydrophilic drugs, hydrophobic drugs, antitumor drugs, siRNA, growth factors and so on (Oss-Ronen and Seliktar, 2011; Baler et al., 2014; Mou et al., 2019). Here, we mainly summarize the application of albumin-based hydrogels in antitumor-drug delivery and tissue regeneration engineering (Figure 2).

### 4.1 Antitumor drug delivery

Like other types of hydrogels for drug delivery, the physical properties of albumin-based hydrogels are highly conducive to drug delivery and enable the sustained release of the encapsulated drug, which can maintain high local drug-concentration over long periods of time through one suitable release mechanism, and the mechanism can be based on diffusion, swelling, chemical or other environmental stimuli (Lantigua et al., 2020; Chen et al., 2021). Injectable hydrogels are often used for controlled and local drug delivery because of their easily adjustable volume, shape, etc (Dimatteo et al., 2018; Rizzo and Kehr, 2021). On the one hand, since the injectable hydrogels are cross-linked *in-situ*, it is more readily to control the gelation time and avoid the shear stress during injection process to affect the performance of hydrogels (Mude et al., 2021). On the other hand, advantages of albumin-based hydrogels as *in situ* injectable hydrogels for antitumor applications are mainly in terms of degradability and morphological modifiability. Zhao et al. constructed a degradable hydrogel for antitumor applications, and this BSA-based hydrogel degrades over time (0–24 h) at 37°C, which indicates that the hydrogel has good degradability. A BSA-based hydrogel with tunable mechanical properties was constructed by Bai et al., which can have a more than 10 kPa storage modulus and compressive stresses up to 0.2 MPa, so this BSA-based hydrogel exhibits good tunability of mechanics and is capable of being injected for biomedical applications (Bai et al., 2019). Cancer therapy is an important application for injectable-polymer hydrogels, and polymeric hydrogels can provide controlled drug-release and targeted therapy, where injectable hydrogels can achieve the effect of non-invasive treatment and monitoring in a single injection, which can bring greater comfort and therapeutic efficacy to patients (Wang et al., 2021). There are differences in the way that albumin-based hydrogels are applied for the treatment of different types of tumor. For example, preventing tumor recurrence of post-surgical glioblastoma could benefit from local hydrogel therapy and so can local immunomodulation for cancer therapy can be achieved with hydrogels (Wang X. et al., 2022). Intermediate to advanced breast cancer is prone to metastasis and spread, hydrogels for *in situ* injection into breast tumor can provide a sustained release of DOX, which effectively inhibits primary regrowth and distant metastases (Qi et al., 2018). During conventional radiation therapy for rectal cancer, the radiation drugs can affect the rectum and cause damage to the rectum. Hydrogel injections *in situ* at the site of prostate cancer can protect the rectum from damage by reducing exposure to chemotherapy drugs (Pinkawa et al., 2016; Yu et al., 2023). For the treatment of osteosarcoma, *in situ* injections of hydrogels loaded with DOX and cisplatin are also better at preventing tumor reoccur or metastasis, while also reducing the systemic toxic effects of the drug (Si et al., 2022). Compared to the four types of cancer (breast cancer, rectal cancer, prostate cancer and osteosarcoma) described above, there are currently fewer cases of injectable hydrogels being used in liver or lung cancer.

In this section, we focus on albumin hydrogels for antitumor drug delivery, and the properties of amyloid fibrils and the advantages of their application in albumin-based hydrogels are also described (Table 2). In general, the tensile strength of albumin-based hydrogels is in the range of tens to hundreds kPa, while their compressive strength is in the range of hundreds kPa to even tens MPa (Zhou et al., 2018; Rong et al., 2022). Albumin-based hydrogels are similar to most protein-based hydrogels in that they have a porous-mesh structure, a high water-retention capacity, a high drug-loading capacity, and the ability to carry various types of drugs (Chander et al., 2021; Kong et al., 2023). Using DOX as the model drug, albumin-based hydrogels were loaded up to ∼0.5 g DOX/g dry-gel in a phosphate buffer at pH 7.4 and ionic strength of 0.01 mol/L, and the albumin-based hydrogel has a drug encapsulation rate of approximately 50%, whose cumulative release rate reached over 90% (Manokruang et al., 2014; Shen et al., 2018; Zhao et al., 2021). In another study, albumin-based hydrogels (BSA) in trypsin medium degraded by ∼50% within 36 h, which proves that they are biodegradable, and DOX release rates within 5 days were ∼37%, ∼26% and ∼21% in PBS solution at pH 5.5, 6.8 and 7.4, respectively (Upadhyay et al., 2018). For all three types of cancer cells (MCF-7, HeLa and MDA-MB-231), the albumin-based hydrogel containing DOX caused about 70%–80% of cell death. Over the last decade, research on albumin-based hydrogels about antitumor has gradually increased (Qian et al., 2019; Chen et al., 2021; Lee et al., 2021). For example, Qian et al. constructed an injectable hydrogel based on albumin, this albumin-based hydrogel has a loading efficiency of 85% and a loading capacity of 22% for paclitaxel. The results of animal studies have shown that this albumin-based hydrogel loaded with paclitaxel has a significant therapeutic effect on gastric cancer with peritoneal metastasis (Qian et al., 2019). The albumin-based hydrogel constructed by Chen et al. has a cumulative release rate of ∼25% DOX within 24 h, and the remaining drug is able to be released sustainably for about 10 days, the hydrogel showed good therapeutic effects for tumor-bearing mice (The highest tumor-inhibition rate of 93.1% was achieved compared to the control group.) (Chen et al., 2021). Lee et al. constructed a HSA-based hydrogel which was effective in inhibiting tumor growth when loaded with hyaluronidase, the tumor volume after treatment with this hyaluronidase-loaded hydrogel was ∼198 mm^3^, while the tumor volume in the control group reached ∼1,230 mm^3^ (Lee et al., 2021). And, the antitumor effect of albumin-based hydrogels is mainly achieved by *in situ* injection of hydrogel. Chen et al. developed an albumin-based injectable hydrogel, and *in vitro* and *in vivo* results showed that the albumin-based hydrogel exhibited significant antitumor efficacy in reducing tumor size (The tumor volume after treatment with this DOX-loading hydrogel was only about 1/6 of the tumor volume in control group.) and appeared to be ideal for local antitumor therapy (Chen et al., 2021). Wang et al. developed a novel hydrogel using BSA and chitosan, the hydrogel has a DOX encapsulation rate of 46.3% and is able to release DOX slowly over a period of 24 h, and cellular uptake experiments showed that it is more efficient in delivering anticancer drugs to tumor compared to bare drugs (Wang et al., 2016). Researchers had also constructed pH-sensitive albumin-based hydrogels using BSA, *ß*-propranolol was rapidly released at pH 1.0 and complete release of *ß*-propranolol was observed after 1 h at pH 6.8 using *ß*-propranolol as a model drug, and it can precisely deliver model drugs to tumor cells (El-Sherif et al., 2009). More information of albumin-based hydrogels in antitumor applications can be found in Table 2.

**TABLE 2:** Applications of albumin-based hydrogels in cancer therapy (Excerpt).

Citations	Types of albumin	Drug	Cancer or tumor cells
Zhao et al. (2019)	BSA	NA	HepG2
Amatya et al. (2021)	BSA	Ag NP	Skin cancer
Chen et al. (2021)	BSA	DOX	4T1
Lee et al. (2022)	BSA	PTX	4T1
Lin et al. (2017)	BSA	PTX	Human brain tumor cells
Qi et al. (2022)	BSA	Photothermal-photodynamic therapy	Breast cancer
Lee et al. (2018)	BSA	DOX	Hypoxic breast cancer
Liu et al. (2018)	BSA	I^131^	Mouse lung cancer cells
Nassar et al. (2019)	BSA	DOX	HepG2
Shen et al. (2018)	BSA	DOX	Drug-resistant breast cancer
Upadhyay et al. (2018)	BSA	DOX	MCF-7, HeLa, MDA-MB-231
Kim et al. (2015)	HSA	TRAIL	MIA PaCa-2
Lee et al. (2020)	HSA	Indocyanine green	Precision cancer surgery
Yamada and Schneider (2016)	BSA	DOX	A549
Lee et al. (2019)	OVA	Anti-tumor vaccines	lymphoma
Li et al. (2022)	BSA	PQ912	Inhibiting local tumor recurrence and distal metastasis
Nandi et al. (2020)	BSA	DOX	A2780, MCF-7, MDA-MB-231, Hela
Noteborn et al. (2017)	HSA	DOX	MCF-7
Yan et al. (2022)	BSA	CDDP, BSO, GOx	A549 and 4T1, cancer combination therapy
Mantooth et al. (2021)	BSA	Immune checkpoint inhibitors	MC38
Qian et al. (2017)	Unclear	PTX	Tumor-regional chemotherapy
Yan et al. (2022)	BSA	Cisplatin	A549 and 4T1

### 4.2 Tissue regeneration

Albumin-based hydrogels are becoming increasingly attractive in tissue engineering as they offer a heterozygous-free, biocompatible and potentially patient-specific platform (Ong et al., 2020). In 2019, Zhang et al. constructed an injectable and rapidly self-healing albumin-based hydrogel, the hydrogel is able to repair itself quickly within 1–2 min with a 100% repair rate. Cytotoxicity assay showed that the survival rate of MCF-7 cells remained about 100% even when the concentration of this hydrogel reached 2%, all above results showed that this albumin-based hydrogel has excellent biocompatibility and has great potential in the field of tissue regeneration engineering (Zhang et al., 2019). Albumin-based hydrogels are used mainly in tissue engineering for several detailed applications, such as wound healing, bone repair, vascular endothelium, scaffold materials and so on. Researchers in Romania constructed a composite hydrogel based on BSA, and the hydrogel showed properties that could be better applied for wound healing (Kenawy et al., 2021). The use of an albumin-based hydrogel (containing BMP2) constructed by Zhang et al. can significantly promote the production of new bone within 13 weeks, the result showed it was effective in accelerating the bridging of tibial bone defects (Kossover et al., 2020). The microporous structure of a photo-cross-linkable HSA hydrogel can be used as a site for host cell infiltration after implantation, the hydrogel can significantly promote the growth of blood vessels (even in 24 h) (Yoon et al., 2021). Table 3 presents more applications of albumin-based hydrogels in tissue regeneration engineering.

**TABLE 3:** Applications of albumin-based hydrogels in tissue regeneration engineering (Excerpt).

Citations	Types of albumin	Detailed application types
Kossover et al. (2020)	BSA	Tibial defect
Claaßen et al. (2017)	BSA	Vascular endothelium
Yoon et al. (2021)	HSA	Vascular endothelium
Layman et al. (2012)	BSA	Vascular endothelium
Areevijit et al. (2020)	BSA	Alveolar bone
Liu et al. (2020b)	BSA	Bone regeneration
Dai et al. (2021)	BSA	Wound healing
Farid et al. (2021)	BSA	Wound healing
Takeda et al. (2015)	BSA	Fibroblasts
Smith et al. (2014)	HSA	Femur defect
Ouyang et al. (2022)	BSA	Wound dressing, the eradication of drug-resistant bacteria
Deng et al. (2022)	BSA	Antibacterial
Lutzweiler et al. (2021)	BSA	Promoting cell proliferation or differentiation
Sharifi et al. (2022)	HSA	Angiogenesis
D'Urso et al. (1995)	BSA	*In vivo* bioreactor
Chen et al. (2019)	BSA	Potential applications in 3D cell culturing or the development of 3D tumor model
Oss-Ronen and Seliktar (2011)	BSA	Tissue-engineered scaffold materials with controlled drug release characteristics
Oss-Ronen and Seliktar (2010)	BSA	Tissue-engineered scaffold materials with controlled drug release characteristics
Berdichevski et al. (2015)	BSA	As scaffold materials in tissue engineering
Patel et al. (2021)	BSA	Subcutaneous drug delivery
Gao et al. (2014b)	BSA	Potentials in biological repair smart materials
Gsib et al. (2020)	BSA	Skin regeneration
Kang et al. (2021)	HSA	Subcutaneous drug delivery
Ribeiro et al. (2016)	BSA and HSA	As scaffold materials in tissue engineering
Liu et al. (2021)	HSA	As a shape-memory material in tissue engineering
Cheng et al. (2020)	BSA	Wound healing

## 5 Potential directions for future research

**FIGURE 3:**
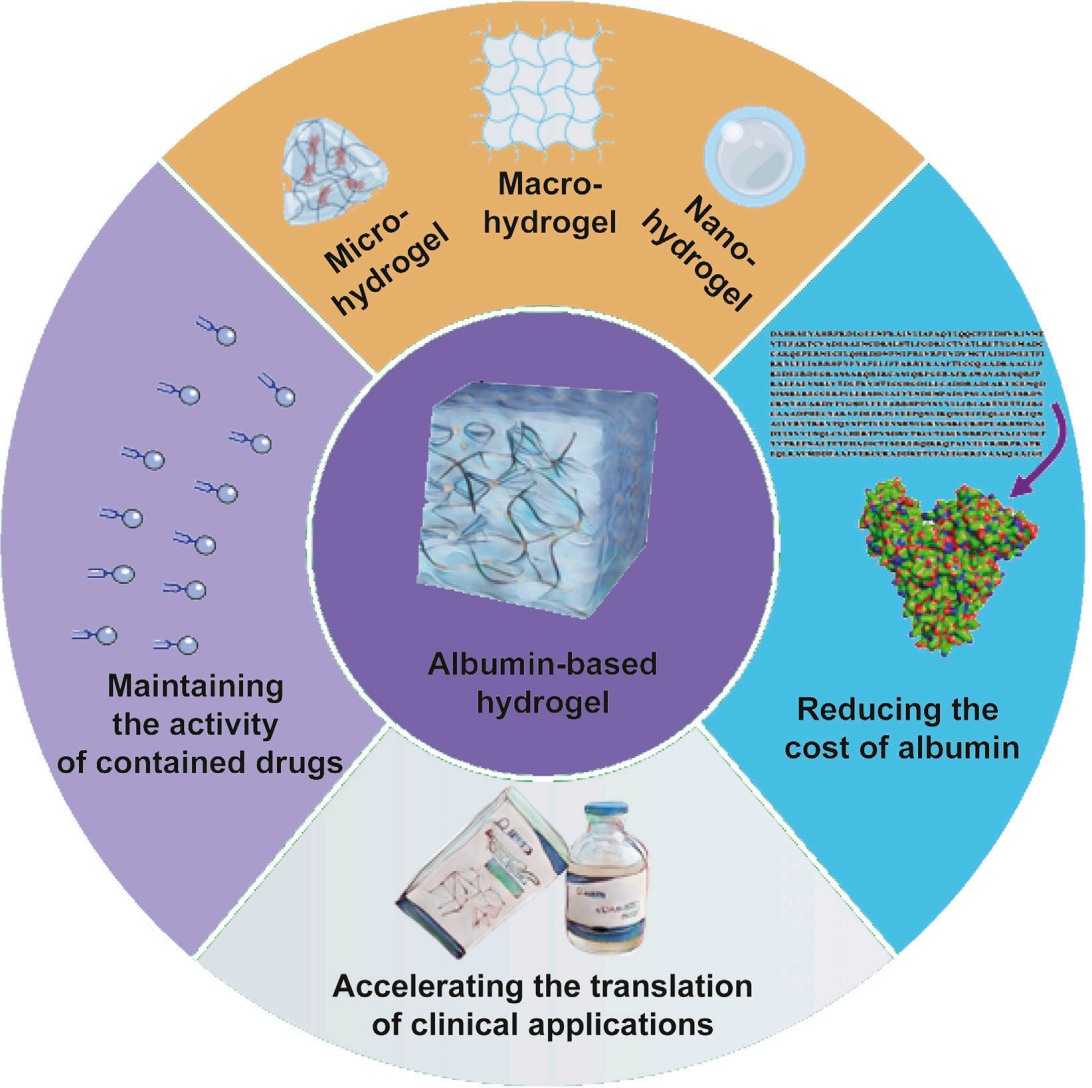
Potential directions for future research of albumin-based hydrogels. There are mainly four potential research directions. Firstly, the activity of drugs, especially biomolecules, such as growth factors, protein inhibitors, etc., should be retained to the maximum extent during the preparation of albumin-based hydrogels. Second, other solutions need to be found to minimize the cost of producing albumin and even preparing albumin-based hydrogels. Such as development of recombinant HSA, enhancing the utilization of ovalbumin, etc. Third, various sizes of albumin-based hydrogels should be developed to meet the needs of clinical applications. The last, the translation of research on albumin-based hydrogels to clinical applications is still rare, and how to make more albumin-hydrogels research translate into practical applications which is one key question that we have to face.

Through the problems we identified during our experiments and our reading of the literature, we think there are four main future research directions for albumin-based hydrogels (Figure 3).

Like other protein-based hydrogels, albumin-based hydrogels are prepared *via* chemical crosslinking, the activity of those biomolecules they carry is also affected because of the use of chemical reagents or heat treatment, pH induction and so on (Zustiak et al., 2013). Therefore, many researchers are trying to improve chemical crosslinking methods for the preparation of hydrogels in hope of retaining the maximum activity of the drugs or biomolecules contained in hydrogels (Oryan et al., 2018; Huang et al., 2021; Zhang et al., 2022). However, our current efforts are still not fully on target, how to maximize the retention of the activity of the contained drugs or biomolecules in the preparation of albumin-based hydrogels and even in the preparation of other hydrogels in the future is an important direction for our future research.

The albumin we currently use to prepare albumin-based hydrogels is mainly derived from human or animal serum, which is relatively expensive and there is also a risk of contamination with various viruses (Chuang and Otagiri, 2007; Mirici-Cappa et al., 2011). Finding solutions to reduce the cost of albumin and the cost of preparing albumin-based hydrogels is a potential direction for our future research. Such as enhancing the research and development of recombinant albumin, improving the utilization of ovalbumin and so on (Rogers et al., 2015). In these alternative solutions, recombinant albumin not only has the advantage of being able to be mass-produced at a lower cost, but also avoids the risk of transmission of blood diseases (Bah et al., 2018; Heroes et al., 2020). A research team from Wuhan University in China successfully used rice to obtain recombinant human serum albumin, and 1 kg rice yields about 10 g HSA, this trial significantly reduced the cost of HSA (He et al., 2011). Currently, a kind of injection prepared from this plant-derived recombinant HSA has already been marketed, and other products prepared from this plant-derived HSA are also entering clinical trials. In 2018, recombinant HSA was obtained by Zhu et al. *via* high-level expression of *pichia pastoris*, and this study showed that *Pichia pastoris* is an excellent system for expressing recombinant HSA (Zhu et al., 2018). The development of two kinds of recombinant HSA could also provide us with a good idea for reducing the cost of albumin and albumin-based hydrogels.

Depending on the size, we can classify hydrogels into 3 categories, namely, macro-hydrogels, micro-hydrogels and nano-hydrogels. Macro-hydrogels are the hydrogels larger than millimetre in size and are usually used for direct injection or implantation around tumor tissue (Sun et al., 2020). Most macro-hydrogels used for cancer therapy are delivered topically. In addition, hydrogels allow for *in situ* sustained release of chemotherapeutics, which increase the solubility and selectivity of drugs and may reduce the total dose of drugs. Currently, the research on albumin-based hydrogels is more on macro-hydrogels. Micro-hydrogels are hydrogels with a size of approximately 0.5–10 μm. Hydrogels of this size have a larger surface area compared to macro-hydrogels and are therefore more suitable for bio-fixation. However, heterologous substances of this size have been reported to be easily engulfed by macrophage and are not suitable for intravascular injection due to the risk of embolization. Therefore, delivery methods of micro-hydrogels for cancer therapy are usually limited to oral delivery, lung delivery or transarterial chemoembolization to treat tumor located in certain organs. Nano-hydrogels are hydrogels less than 200 nm in size. Nanoscale size ensures that nano-hydrogels can bind to target ligands, enhance permeability, enhance retaining effects, target specific tumor and ensure intracellular drug delivery through endocytosis and penetrating the blood-brain barrier. Due to the large surface area, nano-hydrogels can be used for vein injection due to their nanoscale size and have high drug-loading efficiency. Compared to albumin-based macro-hydrogels, there is less research on micro-hydrogels and nano-hydrogels of albumin-based hydrogels (Kong et al., 2023). In the future, it is an important direction to develop various sizes of albumin-based hydrogels to meet the needs of clinical applications.

There is a large body of research on albumin-based hydrogels, but very little of the research has been translated into clinical application. Nowadays, there are many albumin products on the market, such as Abraxane^®^, Fyarro^®^, Idelvion^®^, Tresiba^®^, Victoza^®^, Levemir^®^ and so on (Yardley, 2013; Aberumand and Jeimy, 2021; Li and He, 2022; Pasca and Zanon, 2022; Zhou et al., 2022). But, very few albumin-hydrogel products are listed among them. Why are fewer albumin-hydrogel products currently used in clinical practice? Most hydrogels take a decade to be approved, and have challenges in production and storage, so faster approval is needed and these challenges should be solved. A challenge is that hydrogels contain a large amount of water, and they are subject to breakage during production, so hydrogels are difficult to process in complex or automated ways (Correa et al., 2021). Meanwhile, hydrogels tend to dry out quickly in dry conditions and to freeze in cold climates, which leads to the loss of some of their functions. Thus, one of the challenges facing hydrogels is the storage condition. In brief, how to make these research results of albumin-based hydrogels apply in real life as soon as possible, and even put into mass production with the shortest approval-cycle and the lowest cost is an important research direction that we should dive in the future.

## 6 Conclusion

Albumin-based hydrogels have some better advantages such as controlled mechanical properties, better biocompatibility, better degradability and so on, which has made albumin-based hydrogels attract the attention of an increasing number of researchers. Currently, albumin-based hydrogels have made significant progress in antitumor-drug delivery and tissue regeneration engineering. However, there are still four main issues waiting to be addressed. The first is how to maintain the activity of drugs including biomolecules during the preparation of albumin-based hydrogels. Secondly, more albumin-based hydrogels of different sizes should be developed to meet the needs of practical applications. Thirdly, at present, most of albumin used in the preparation of albumin-based hydrogels is derived from human or animal serum, and it is a question of whether other ways can be explored to reduce the cost and the use of serum albumin. For example, exploring mass-production of recombinant albumin or increasing the utilization of ovalbumin. Last but not least, how to translate these research results into clinical applications as soon as possible remains as an urgent problem needs to be solved.

## Abbreviations

BSA, Bovine serum albumin; HSA, Human serum albumin; JTA, Janus Tough Adhesive; kDa, Kilo Dalton; CMPF, 3-Carboxy-4-methyl-5-propyl-2-furanpropionic acid; MRI, Magnetic resonance imaging; siRNA, Small interfering RNA; NA, Not applicable; Ag NP, Silver nanoparticle; HepG2, Human hepatoellular carcinomas; DOX, Doxorubicin; PTX, Paclitaxel; 4T1, Mouse breast cancer cells; MCF-7, Human breast cancer cell line; HeLa, Hela Cells; MDA-MB-231, Human breast cancer cells; TRAIL, TNF-related apoptosis inducing ligand; MIA, PaCa-2 Human pancreatic cancer cells; A549, Human Non-Small Cell Lung Cancer Cells; PQ912, it is a Glutaminyl Cyclase (QCPT) inhibitor; A2780, Ovarian carcinoma; CDDP, Cisplatin; BSO, L-Buthionine-sulfoximine; GOx, Glucose oxidase; MC38, Mouse colon cancer cell line; APS, Ammonium persulfate; FAs, fatty acids.
